# Hemodynamic and Light-Scattering Changes of Rat Spinal Cord and Primary Somatosensory Cortex in Response to Innocuous and Noxious Stimuli

**DOI:** 10.3390/brainsci5040400

**Published:** 2015-09-29

**Authors:** Ji-Wei He, Hanli Liu, Yuan Bo Peng

**Affiliations:** 1 Departments of Psychology, University of Texas at Arlington, Arlington, TX 76019, USA; E-Mail: ypeng@uta.edu; 2 Department of Neurological Surgery, University of California San Francisco, 1700 Owens Street, San Francisco, CA 94158, USA; 3 Department of Bioengineering, University of Texas at Arlington, Arlington, TX 76019, USA; E-Mail: hanli@uta.edu

**Keywords:** pain, oxygenated hemoglobin, deoxygenated hemoglobin, oxygen saturation, neurovascular coupling, light scattering

## Abstract

Neuroimaging technologies with an exceptional spatial resolution and noninvasiveness have become a powerful tool for assessing neural activity in both animals and humans. However, the effectiveness of neuroimaging for pain remains unclear partly because the neurovascular coupling during pain processing is not completely characterized. Our current work aims to unravel patterns of neurovascular parameters in pain processing. A novel fiber-optic method was used to acquire absolute values of regional oxy- (HbO) and deoxy-hemoglobin concentrations, oxygen saturation rates (SO_2_), and the light-scattering coefficients from the spinal cord and primary somatosensory cortex (SI) in 10 rats. Brief mechanical and electrical stimuli (ranging from innocuous to noxious intensities) as well as a long-lasting noxious stimulus (formalin injection) were applied to the hindlimb under pentobarbital anesthesia. Interhemispheric comparisons in the spinal cord and SI were used to confirm functional activation during sensory processing. We found that all neurovascular parameters showed stimulation-induced changes; however, patterns of changes varied with regions and stimuli. Particularly, transient increases in HbO and SO_2_ were more reliably attributed to brief stimuli, whereas a sustained decrease in SO_2_ was more reliably attributed to formalin. Only the ipsilateral SI showed delayed responses to brief stimuli. In conclusion, innocuous and noxious stimuli induced significant neurovascular responses at critical centers (e.g., the spinal cord and SI) along the somatosensory pathway; however, there was no single response pattern (as measured by amplitude, duration, lateralization, decrease or increase) that was able to consistently differentiate noxious stimuli. Our results strongly suggested that the neurovascular response patterns differ between brief and long-lasting noxious stimuli, and can also differ between the spinal cord and SI. Therefore, a use of multiple-parameter strategy tailored by stimulus modality (brief or long-lasting) as well as region-dependent characteristics may be more effective in detecting pain using neuroimaging technologies.

## 1. Introduction

While decades of discoveries using neuroimaging technologies have revealed rich and complex processes underlying various brain functions [1], the use of neuroimaging as an objective tool to quantify or measure pain has been questioned [2]. This is because pain is a multifactorial subjective experience of the nociceptive inputs associated with one’s memories as well as emotional, pathological, genetic, and cognitive factors [3]. The often-called “pain matrix” due to a large distributed brain network involved during nociceptive/pain processing leads to limited validation and effectiveness (as examined by sensitivity and specificity) of neuroimaging signals in pain detection and/or quantification [4].

In order to validate, improve or support neuroimaging as an effective and objective tool to measure pain, a better understanding of neural, cerebral, and/or vascular physiology under different kinds of painful/noxious stimulation and at different locations of the central nervous system would be beneficial. A few recent studies using functional magnetic resonance imaging (fMRI) have confirmed that BOLD signals, originating from regional deoxy-hemoglobin concentration changes, are altered due to thermal pain and can be used as biomarkers to objectively assess pain [5,6]. Similarly, several human studies using near infrared spectroscopy have demonstrated that thermal or electrical stimulations induce changes of hemoglobin concentrations at different brain regions [7,8,9]. However, many important physiological questions related to pain processing at different neurological sites cannot be answered using a non-invasive approach in human subjects. Animal studies become a necessary and important approach to address many of the physiological questions. In this particular study, we aimed to answer the following questions: (i) how vascular hemoglobin concentrations and oxygenation change under various short-term mechanical and electrical stimuli as well as a long-lasting chemical stimulus; (ii) whether the nociception-induced hemoglobin-based parameters are contralateral or bilateral in the primary somatosensory cortex (SI) and/or spinal cord; and (iii) whether there exists a single parameter that is consistently associated with noxious stimuli.

To answer the questions given, it is necessary to precisely characterize the neurovascular coupling during pain processing in the central nervous system. In this study, we utilized our recently-developed fiber-optic method [10,11] that allows us to simultaneously acquire absolute values of regional oxy-hemoglobin and deoxy-hemoglobin concentrations (*i.e.*, HbO and Hb) as well as the light-scattering coefficients (μ_s_′: an effect describing photons being dispersed when diffusing into biological tissue) from rat bilateral spinal cord and SI. In particular, the light-scattering property of neurons is believed to manifest neural activation in various species, from the crab leg-nerves to squid giant-axons [12,13], and from the retina to neocortices in cats and rats [14,15,16,17,18,19]. To investigate the effectiveness of our optical signals-derived neurovascular parameters in pain detection/quantification, we used three different stimulus modalities, namely graded mechanical (brushing, innocuous pressuring, and noxious pinching), graded electrical (5, 10, and 15 V), or long-lasting chemical stimulus (formalin injection) to rat hindpaw. Our results indicated that all neurovascular parameters showed stimulation-induced changes. However, patterns of these stimulation-induced changes varied with regions and stimuli, suggesting that a multiple-parameter strategy tailored with respect to stimulus modality may be more effective in pain detection/quantification.

## 2. Materials and Methods

### 2.1. Animal Preparation

Ten male adult Sprague-Dawley rats were used with a mean age of 102.1 ± 0.6 (±SEM) days and a mean weight of 377.9 ± 14.5 g. All animals were initially anesthetized by a single intraperitoneal injection of pentobarbital sodium solution (50 mg/kg). A PE10 tubing was inserted into the jugular vein for continuous intravenous (i.v.) administration of pentobarbital sodium (5 mg/mL) at a fixed rate of 0.02 mL/min to maintain anesthesia throughout data acquisition [20]. The lumbasacral segment of rat spinal cord was exposed following laminectomy and then animal was immobilized on a stereotaxic frame. The dura mater was resected, and mineral oil was used to cover the spinal cord to preserve moisture. Rat body temperature was maintained at 37 °C by using a feedback-controlled heating blanket (Homoeothermic Blanket, Harvard Apparatus, Holliston, MA, USA). The animal was further paralyzed by i.v. injection of pancuronium (1 mL; 1 mg/1 mL/min) to prevent muscular twitches. Artificial ventilation (Model 683, Harvard Apparatus, Holliston, MA, USA) was maintained throughout the experiment. All procedures were approved by the Institutional Animal Care and Use Committee (IACUC) at the University of Texas at Arlington. Procedures also followed the guidelines described by the Committee for Research and Ethical Issues of the International Association for the Study of Pain [21].

### 2.2. Data Collection

A customized needle-like fiber-optic system was used to collect reflectance of light at wavelengths between 400 and 1000 nm [11]. Prior to the placement of optic probes over the spinal cord, a silver ball-electrode was used to locate the ipsilateral dorsal root entry zone where strength of the primary afferent inputs was maximal by gently tapping rat hindpaw and monitoring real-time recording (using a oscilloscope and an audiometer). Two craniotomies were made over the bilateral primary somatosensory cortices for hindlimb at posterior AP 0.8 mm and ±2 mm lateral, and burr holes were then filled with drops of mineral oil before the placement of optic probes to avoid air gaps. Four optic probes (0.85 mm in diameter) were positioned at the dorsal surface of the dorsal root entry zones at L4-5 lumbar segment, and at SI burr holes bilaterally. A surgical microscope (Zeiss) was used to ensure no-pressure contacts of probes to the SI and spinal cord. To investigate the microcirculation system during functional activation, we purposely avoided large vessels for probe placements. The use of bilateral assessments (of the spinal cord and SI) allowed us to confirm functional activation during sensory processing. After the experiment, optical signals were converted into HbO, Hb, and μ*_s_*′ by using an iterative algorithm in Matlab (MathWorks, Natick, MA, USA). The algorithm is detailed elsewhere [11]. The sampling rate was 0.6 Hz.

### 2.3. Mechanical, Electrical and Chemical Stimuli

Graded mechanical (e.g., brushing, pressuring and pinching) stimuli and electrical (e.g., 5, 10, and 15 V) stimuli at 10 Hz with 1-millisecond pulse-duration (Grass S48 stimulator, USA) were applied to rat hindlimb unilaterally for 10 s. Mechanical stimuli were applied on the plantar surface [22,23]. Electrical stimuli were delivered to the ankle using two leads (*i.e.*, bent syringe-needles) pierced through the skin. Both mechanical and electrical stimulations were conducted in a block design with five consecutive trials and an interval of 2 min to the same paw for each animal. After mechanical and electrical stimulation, formalin (50 μL; 3%) was injected into the center of the plantar area of the other paw. Some data from electrical stimulation paradigm were reported elsewhere [11].

### 2.4 Statistical Analysis

Wilks’ lambda, a multivariate Analysis of Variance (ANOVA), was utilized to test a baseline difference between the spinal cord and SI based on bilateral measurements in each parameter (e.g., HbO, Hb, HbT, SO_2_ or μ_s_′). *T-*tests were utilized to test a stimulation-induced relative change from baseline or to test a difference between hemispheres. Univariate one-way within-subject ANOVA was used to test formalin-induced relative changes over time. *Post-hoc* contrast analysis and Fisher LSD multiple comparisons were conducted to reveal a temporal pattern of formalin-induced change if necessary. The alpha level was set at 0.05. All data were expressed in mean ± SEM. All statistical analyses were performed in SPSS 17.0 (SPSS, Chicago, IL, USA).

## 3. Results

### 3.1. Basal Levels of Hemodynamic Parameters and Light-Scattering Coefficients

Table 1 illustrates baseline measurements of HbO, Hb, HbT (HbO+Hb), SO_2_ (HbO/HbT), and μ_s_′ before the first stimulation, brushing, at four locations (*n* = 10). Within the spinal cord or SI, there appeared no systemic difference between hemispheres. Between the spinal cord and SI, there were significant differences in HbO, SO_2_, HbT and μ_s_′. Most prominently, the basal HbO in the SI was higher than that in the spinal cord, whereas the basal light-scattering in the SI was lower than that in the spinal cord. These basal differences should reflect a structural discrepancy. Anatomically, the scanned region at the SI was mainly the grey matter, whereas at the spinal cord it was mainly the white matter.

**Table 1:** Absolute values of baseline neurovascular parameters (*n* = 10).

	Spinal Cord		SI		SP *vs* SI
	Ipsi	Cont	Ipsi	Cont	
**HbO** (µM)	31.1 ± 18.7	14.5 ± 4.5	112.1 ± 21.7	71.8 ± 15.6	*p* = 0.002 (*)
**Hb** (µM)	50.5 ± 28.7	18.8 ± 7.9	46.7 ± 16.7	68.1 ± 18.9	*p* = 0.079
**SO_2_**	0.40 ± 0.07	0.52 ± 0.07	0.70 ± 0.08	0.51 ± 0.07	*p* = 0.044 (*)
**HbT** (µM)	81.3 ± 47.0	33.4 ± 11.0	160.1 ± 24.7	140.0 ± 30.7	*p* = 0.014 (*)
**µ_s_′** (cm^-1^)	29.5 ± 3.6	35.1 ± 2.8	13.5 ± 2.9	11.5 ± 0.6	*p* < 0.001 (*)

*: *p* < 0.05 between the spinal cord and primary somatosensory cortex for hindlimb (SI). Ipsi: ipsilateral. Cont: contralateral. SP: the spinal cord. Mean ± SEM.

### 3.2. Mechanical- and Electrical-Stimulation-Induced Hemodynamic and Light-Scattering Changes

**Figure 1:**
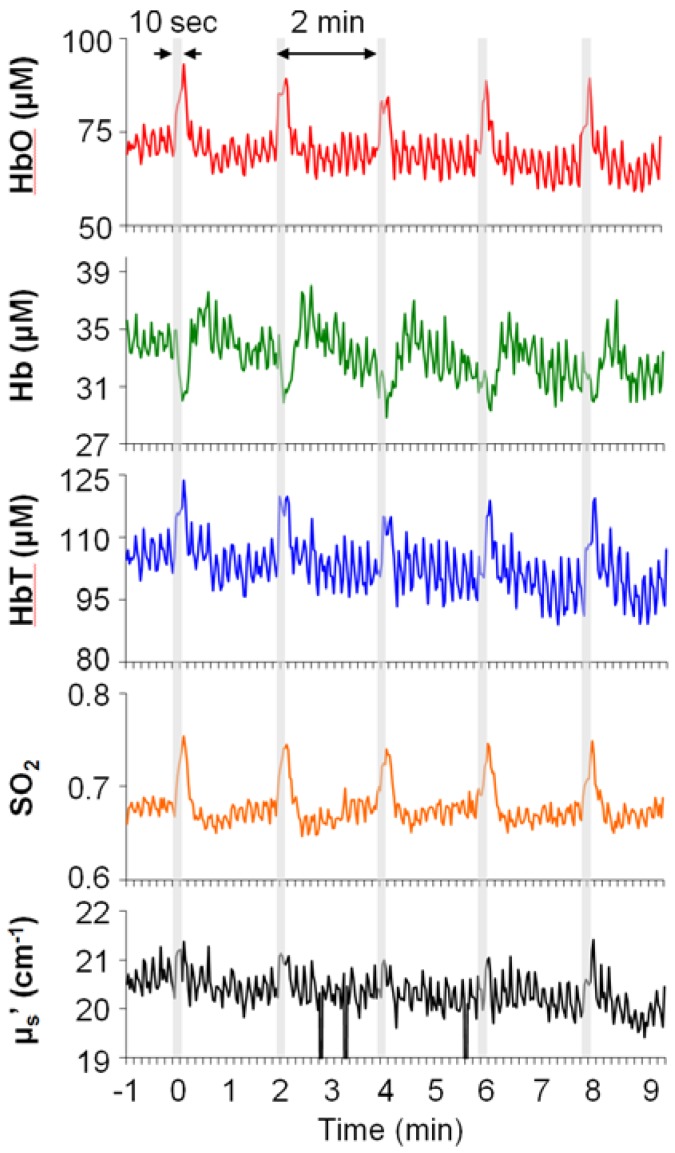
Typical examples of neurovascular responses to electrical stimulation of rat hindlimb from the ipsilateral spinal cord. Stimulation parameters: 10 s; 15 V; 10 Hz pulse train; 1-millisecond pulse-duration. Grey bars: five blocks of stimulation windows.

**Figure 2:**
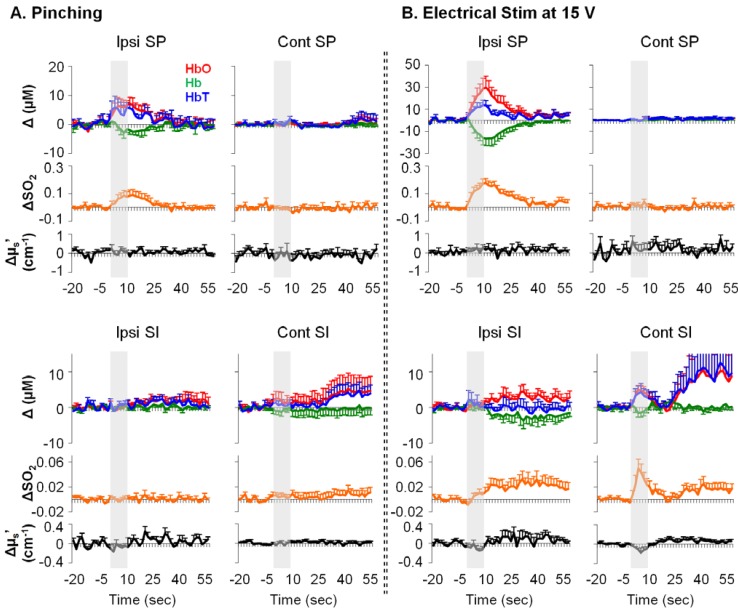
Examples of relative changes in neurovascular parameters from rat spinal cord and SI in response to noxious pinching (**A**) and noxious electrical stimulation ((**B**); *n* = 10). Ipsi/cont: ipsilateral/contralateral; SP: the spinal cord. Error bars: SEM. Grey bars: 10-s stimulation period. Δ: relative change from baseline.

Typical examples of stimulation-induced hemodynamic and light-scattering traces from the ipsilateral spinal cord are shown in Figure 1. Examples of relative changes (after block averaging, *i.e.*, five blocks, and baseline subtraction) are shown in Figure 2. In particular, the ipsilateral spinal cord tended to show longer (~30 s) responses in HbO, Hb, HbT and SO_2_, whereas the contralateral SI tended to show shorter (~10 s) responses in HbO, HbT, SO_2_, and μ_s_′.

**Figure 3:**
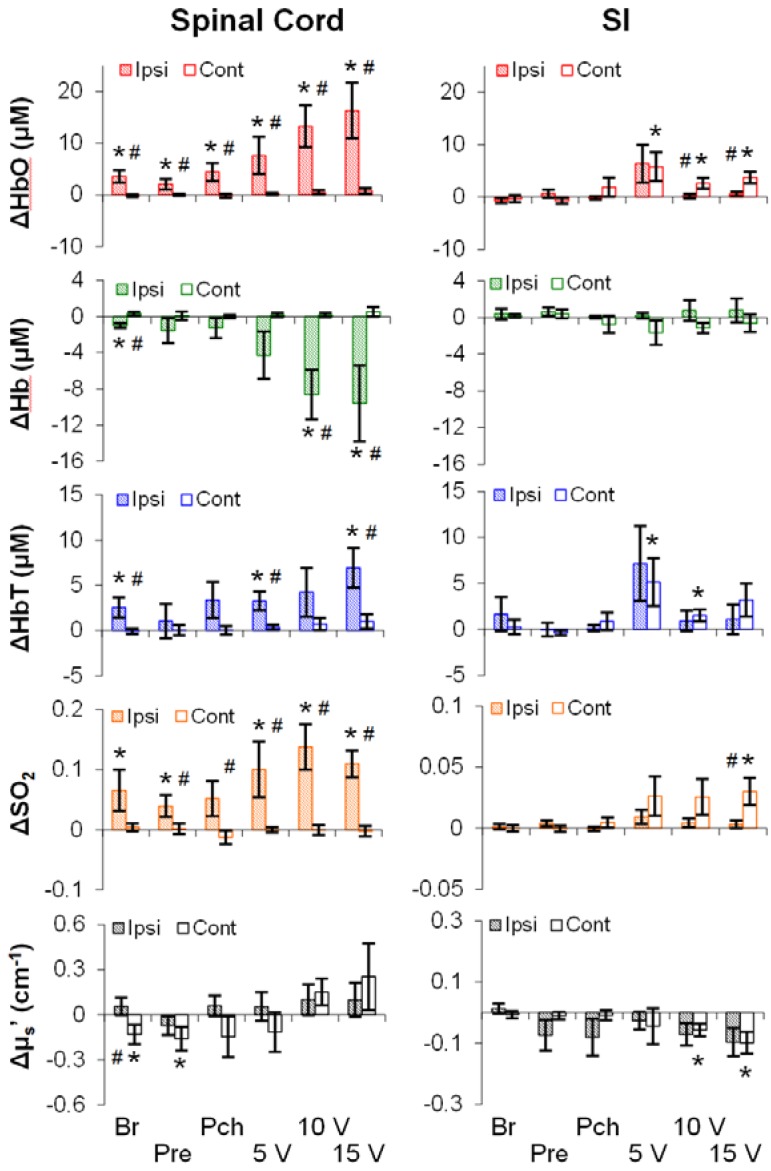
Relative changes in neurovascular parameters in response to mechanical and electrical stimulations (*n* = 10). Shaded/empty bars: ipsilateral/contralateral side. #: *p* < 0.05 between sides; *: *p* < 0.05 against 0. Error bars: SEM. Br: brushing. Pre: pressuring. Pch: pinching.

To investigate the effects of stimulus modality (mechanical or electrical), intensity (low, medium, or high), and region (the SI or the spinal cord) on hemodynamics as well as on the light scattering, averages of representative time periods (30 s for the spinal cord; 10 s for the SI) were used. As shown in Figure 3, all five parameters (rows) showed stimulation-induced changes along the somatosensory pathway (*i.e.*, the ipsilateral spinal cord and/or the contralateral SI). Surprisingly, the stimulation-induced changes did not always occur even at the highest intensity of stimulation. That is, the stimulation-induced changes were not merely dependent on intensity, but on other factors such as region and modality. For instance, the ipsilateral spinal cord was responsive to both modalities, whereas the contralateral SI was responsive to only electrical stimuli. In addition, the spinal cord was more likely to show a change in Hb than the SI. Finally, both regions showed a decrease in μ_s_′, despite no consistency in intensity, modality or lateralization.

### 3.3. Temporal Characteristics of Hemodynamics in the Spinal Cord and SI

**Figure 4:**
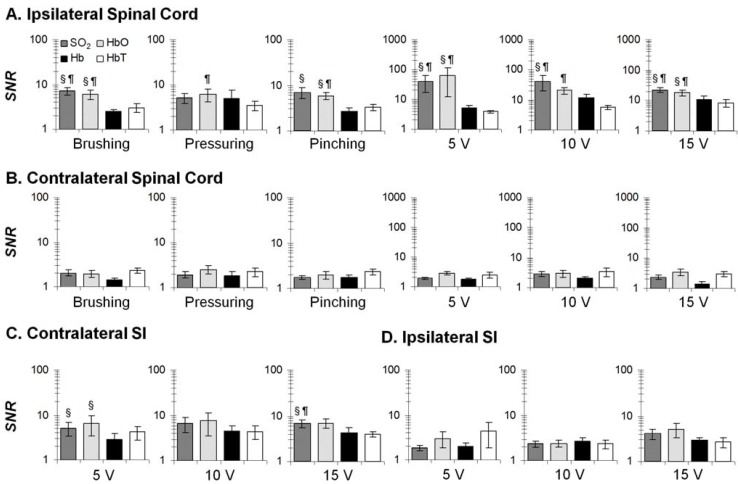
*SNR* of hemodynamic parameters (*n* = 10) at four conditions: ipsilateral spinal cord (**A**); contralateral spinal cord (**B**); contralateral SI (**C**); and ipsilateral SI (**D**). Four comparisons: SO_2_ *vs.* Hb; SO_2_ *vs.* HbT; HbO *vs.* Hb; HbO *vs.* HbT. §: *p* < 0.05 against Hb; ¶: *p* < 0.05 against HbT. Error bars: SEM.

To precisely investigate temporal profiles of hemodynamic changes in response to brief stimuli, we sought to determine the most reliable hemodynamic parameter. A signal-to-noise ratio (*SNR*; the maximum divided by the standard deviation of 20-s baseline measurement) of HbO, Hb, HbT, and SO_2_ were computed; and the parameter with the largest *SNR* indicating the most reliable parameter. Our *SNR* essentially utilized the peak value of a neurovascular response that was intended to minimize the complex temporal dynamics. As shown in Figure 4, SO_2_ and HbO were generally superior to Hb and HbT across regions, stimulus intensities and modalities. These two were selected for temporal analysis.

As shown in Table 2, onset, peak time and duration (all in seconds) were used to demonstrate temporal characteristics of stimulation-induced changes in HbO and SO_2_. Note that only a fraction of rats showed a detectable onset at all stimulation conditions. To maintain sufficient statistical power, no statistical analysis was performed for less representative cases (*n* < 5). In brief, the ipsilateral spinal cord response appeared to occur later than the contralateral SI response (~4 *vs.* ~2 s in SO_2_; noted by †). Also, the ipsilateral spinal cord responses peaked later than the contralateral SI responses (~11 *vs.* ~4 s in HbO or SO_2_; noted by †). Similar to the peak time, the ipsilateral spinal cord responses returned to baseline later than the contralateral SI response (duration: ~30 *vs.* ~8 s in HbO or SO_2_; noted by †). Finally, there were delayed ipsilateral SI responses (~15 s in HbO or SO_2_; noted by ‡). Collectively, there were significant differences in temporal profile of stimulation-induced changes between the spinal cord and the SI.

**Table 2:** Onset, peak time and duration (all in seconds) of stimulation-induced changes in HbO (left) and SO_2_ (right).

		HbO				SO_2_			
		Onset	Peak	Duration	*n*	Onset	Peak	Duration	*n*
**Ipsi SP**	**Br**	**4.8 ± 1.1 (‡)**	9.8 ± 0.9	15.6 ± 3.1	8	7.3 ± 1.6	13.8 ± 1.4	19.5 ± 3.6	10
	**Pre**	4.6 ± 1.4	13.7 ± 2.6	17.4 ± 4.0	9	5.0 ± 1.0	13.7 ± 2.6	17.2 ± 3.7	9
	**Pch**	**4.0 ± 1.0 (‡)**	10.0 ± 1.1	15.6 ± 3.3	8	3.5 ± 0.5	13.1 ± 1.8	19.8 ± 3.2	8
	**5V**	**3.2 ± 0.7 (‡)**	**10.8 ± 0.9 (†)**	**23.5 ± 4.1 (†)**	10	**4.2 ± 0.7 (‡†)**	**12.3 ± 0.8 (†)**	**30.8 ± 4.5 (†)**	10
	**10V**	**4.0 ± 1.0 (‡)**	**11.5 ± 0.9 (†)**	**29.2 ± 3.8 (†)**	10	**2.8 ± 0.5 (‡)**	**11.0 ± 0.4 (†)**	**35.3 ± 3.8 (†)**	10
	**15V**	3.8 ± 1.5	**12.3 ± 1.1 (†)**	**33.7 ± 5.2 (†)**	10	**4.2 ± 1.5 (‡†)**	**13.3 ± 1.0 (†)**	**30.7 ± 6.2 (†)**	10
**Cont SI**	**Br**	2.2 ± 1.5	5.0 ± 1.9	15.0 ± 13.3	3	3.3 ± 1.7	3.3 ± 1.7	2.5 ± 0.8	2
	**Pre**	5.0 ± 3.3	5.0 ± 3.3	1.7 ± 0	2	8.3	8.3	1.7	1
	**Pch**	4.2 ± 4.2	6.7 ± 1.7	4.2 ± 2.5	2	2.8 ± 2.8	4.4 ± 2.4	3.3 ± 1.7	3
	**5V**	1.9 ± 0.8	4.1 ± 1.1	± 2.1	7	**1.1 ± 0.6 (‡)**	4.4 ± 1.2	9.2 ± 2.3	6
	**10V**	**3.3 ± 0.7 (‡)**	4.8 ± 0.7	4.5 ± 1.6	7	2.5 ± 0.5	4.0 ± 0.6	5.8 ± 1.7	8
	**15V**	2.9 ± 0.9	5.4 ± 0.8	13.8 ± 4.6	8	**1.9 ± 0.5 (‡)**	3.7 ± 0.2	10.4 ± 2.1	9
**Ipsi SI**	**Br**	23.8 ± 6.2	38.3 ± 9.1	19.0 ± 8.7	8	15.3 ± 7.3	33.1 ± 12.3	22.8 ± 10.8	6
	**Pre**	9.8 ± 3.1	21.9 ± 9.8	19.5 ± 9.1	7	13.7 ± 9.7	33.0 ± 13.4	24.0 ± 15.9	5
	**Pch**	27.4 ± 9.5	47.6 ± 8.4	18.8 ± 5.6	7	22.8 ± 8.5	52.5 ± 12.7	32.8 ± 11.6	6
	**5V**	20.2 ± 8.8	30.5 ± 6.7	21.4 ± 12.1	7	23.3 ± 6.5	35.4 ± 5.8	16.3 ± 9.5	8
	**10V**	15.0 ± 4.4	33.0 ± 5.4	26.5 ± 5.9	9	15.6 ± 5.2	26.7 ± 6.4	13.2 ± 4.0	9
	**15V**	7.6 ± 2.6	23.5 ± 7.5	21.7 ± 8.8	9	18.5 ± 6.1	29.8 ± 6.2	31.3 ± 9.8	8

Onset: the first time point since stimulation start when *Z-score* (HbO or SO_2_ divided by the standard deviation of 20-s baseline) > 1.96. Peak: the time point of the maximum since stimulation start. Duration: the period between the onset and the first time point when *Z-score* < 1.96. Sample size (*n*): number of rats with a valid onset. †: *p* < 0.05 between the ipsilateral spinal cord and the contralateral SI; ‡: *p* < 0.05 between the ipsilateral SI and the ipsilateral spinal cord (or the contralateral SI).

### 3.4. Formalin-Induced Hemodynamic and Light-Scattering Changes

**Figure 5:**
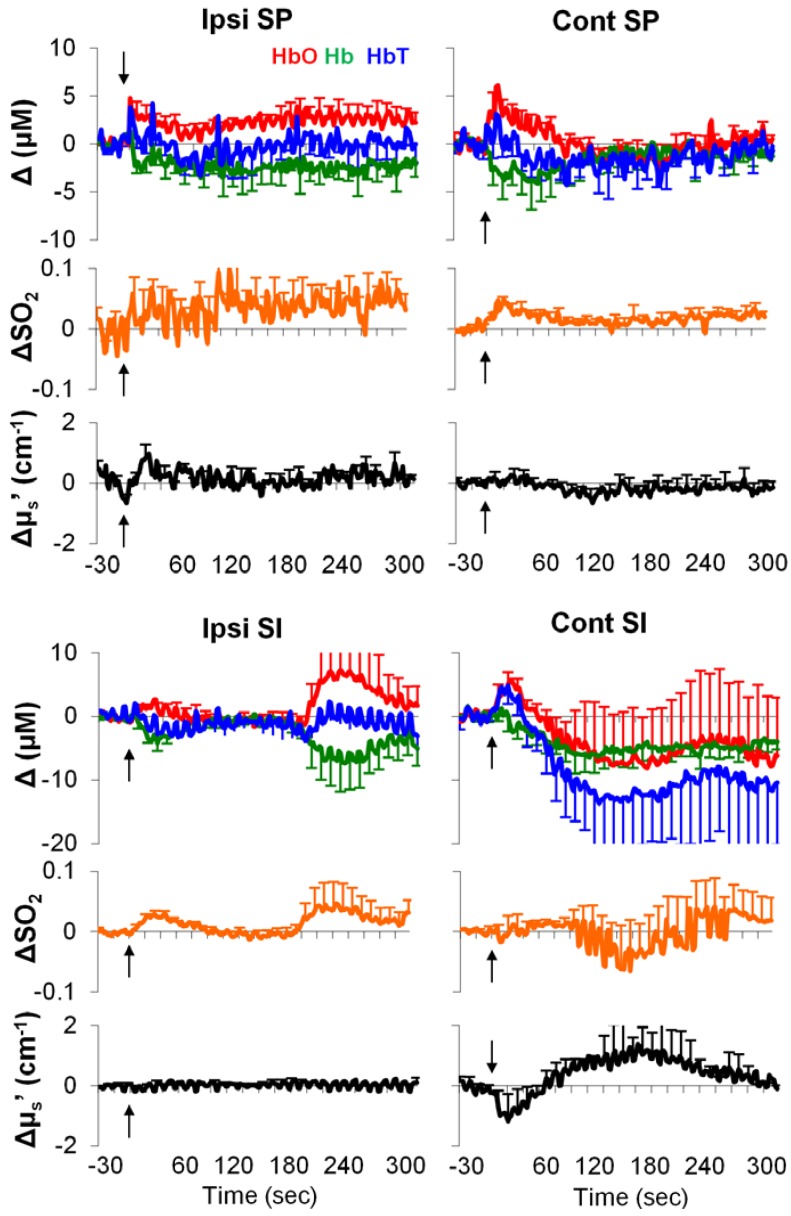
Acute neurovascular responses to formalin injection. Values: relative changes from baseline. Black arrows: injection time. Error bars: SEM.

As shown in Figure 5, in the acute phase, the formalin-induced responses appeared to be highly variable over time. Averages of the first 60-s period indicated the following: (1) an increase in HbO and a decrease in Hb from the ipsilateral spinal cord and SI; (2) an increase in SO_2_ from the contralateral spinal cord and the ipsilateral SI; (3) a decrease in HbT only from the ipsilateral SI (Table 3). In the acute phase, we failed to find any statistically significant change from the contralateral SI. This may in part be due to a considerable level of individual difference (see large error-bars in Figure 5). Note that due to massive bleedings and an unexpected death, the sample size differed among regions (for the bilateral SI and contralateral spinal cord: *n* = 8; for the ipsilateral spinal cord: *n* = 9).

**Table 3:** Acute neurovascular responses to formalin injection. Values: relative changes from baseline.

	Ipsi SP	Cont SP	Ipsi SI	Cont SI
**ΔHbO** (μM)	2.2 ± 0.8 (*)	2.5 ± 1.8	0.8 ± 0.3 (*)	2.1 ± 1.3
**ΔHb** (μM)	−1.6 ± 0.7 (*)	−2.8 ± 1.8	−2.3 ± 0.6 (*)	−1.5 ± 1.3
**ΔSO_2_**	0.02 ± 0.03	0.03 ± 0.007 (*)	0.02 ± 0.002 (*)	0.003 ± 0.1
**ΔHbT** (μM)	0.3 ± 0.5	−0.1 ± 0.8	−1.3 ± 0.6 (*)	0.5 ± 2.2
**Δµ_s_′** (cm^−1^)	0.3 ± 0.2	0.02 ± 0.2	−0.002 ± 0.02	−0.5 ± 0.5

*: *p* < 0.05 against 0. Average period: 0–60 s.

**Figure 6:**
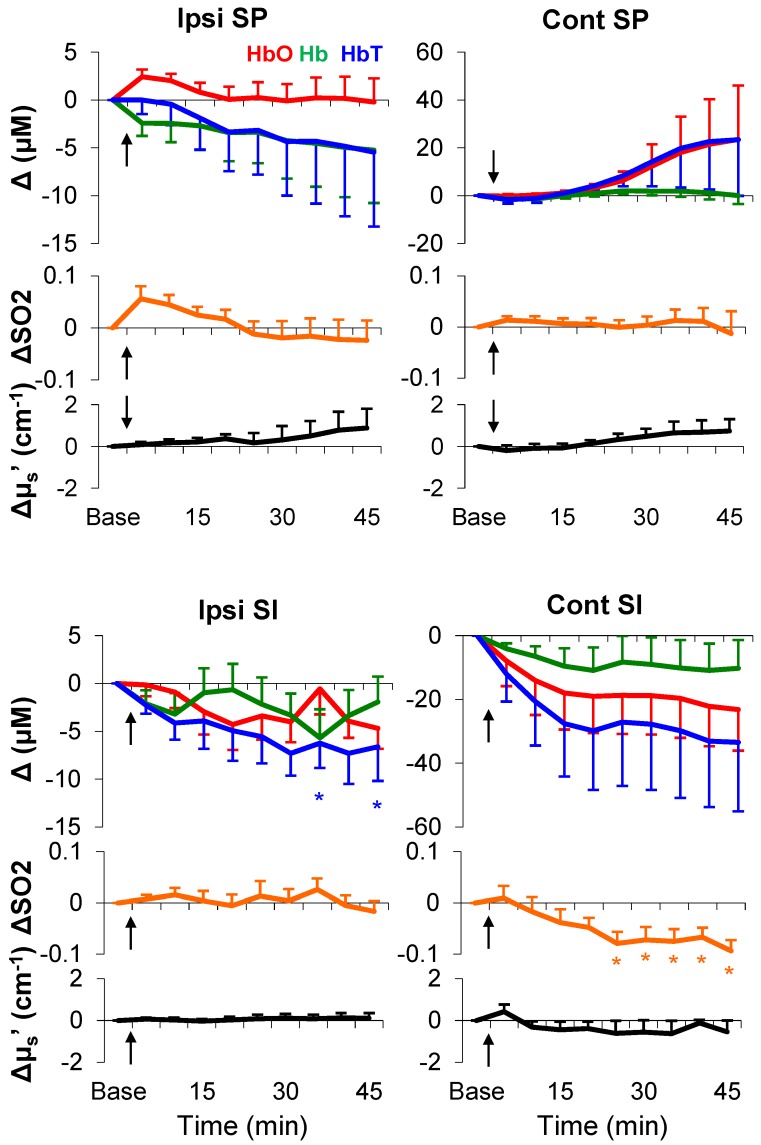
Full time course of neurovascular responses after formalin injection. Values: relative changes from baseline. Black arrows: injection time. *: *p* < 0.05 (*post-hoc* Fisher LSD). Each time point: average of 5 min. Error bars: SEM.

As shown in Figure 6 and Table 4 (see averages of every 5 min over a total of 45 min), in the delayed phase, only sustained decreases in SO_2_ (from the ipsilateral spinal cord and the contralateral SI), HbO (only from the contralateral SI), and HbT (from bilateral SI) were statistically significant. It is noteworthy that the light scattering failed to show any statistically significant change after formalin injection. Together, the hemodynamic parameters, namely SO_2_, HbO, and HbT, demonstrated formalin-induced changes; patterns of these changes were functions of time and region.

**Table 4:** *P*-values of time effect on neurovascular parameters.

	Ipsi SP	Cont SP	Ipsi SI	Cont SI
**SO_2_**	0.024 (^a,^*)	0.98	0.61	0.001 (^a,^*)
**HbO**	0.48	0.38	0.08	0.012 (*)
**Hb**	0.71	0.33	0.40	0.45
**HbT**	0.79	0.33	0.046 (^a, ^*)	0.046 (*)
**μ_s_′**	0.80	0.35	0.98	0.35

*: *p* < 0.05 main effect; ^a^: *p* < 0.05 *post-hoc* contrast analysis on linearity.

## 4. Discussions

We measured focal hemodynamic and light-scattering changes following mechanical, electrical, and chemical noxious stimulation in rat SI and spinal cord by using a fiber-optic method. A phantom experiment with a similar method suggests a signal penetration depth to be ~1 mm [19]. Thus, our measurements in rats should have included sizable regions for accessing functional activation of the SI and spinal cord. Our absolute measurements of HbT from the SI are in a physiological range of a previous report [24]. More importantly, our results of electrical stimulation-induced HbO and Hb changes in opposite directions from the ipsilateral spinal cord and the contralateral SI are well-documented and are in agreement with the somatosensory pathway, suggesting that our measurements are functionally relevant to neural activation. In contrast to the contralateral SI, the ipsilateral SI, to a lesser extent, is thought to play a role in somatosensory processing as examined by human neuroimaging techniques [25,26,27], likely via the corpus callosum [28]. The ipsilateral SI’s neural activity also occurs later, and is weaker in intensity [27]. Regarding onset and intensity, our results (Figure 2 and Table 2) appeared to resemble the electrophysiology of the human brain. Nevertheless, the physiological significance of the ipsilateral SI in somatosensory processing or pain processing remains poorly understood.

### 4.1. Electrical, but Not Mechanical, Stimuli Produced an Intensity-Dependent HbO Increase

The essence of our work was to determine a biomarker of pain. As shown in Figure 3, using HbO or any other parameter alone was unlikely to differentiate noxious stimuli (pitching or 15 V) from innocuous stimuli (brushing or 5 V). However, HbO from the spinal cord showed an intensity-dependent change in response to electrical stimuli (*F*(2, 18) = 4.15, *p* = 0.03), but not to mechanical stimuli (*F*(2, 18) = 1.85, *p* = 0.19). This modality disparity may be related to the nature of stimuli, but not nociception.

In the peripheral nervous system, the primary afferents respond differently to various stimuli in intensity- and modality-dependent manners. Modality is defined as a general type of stimulus that is associated with a specific type of receptor on the primary afferents [29]. As an innocuous stimulus, brushing only activates A-β primary afferent fibers conveying information from mechanoreceptors, whereas noxious pinching primarily activates A-δ and C fibers conveying information from both mechanoreceptors and nociceptors [30]. As an unnatural stimulus, electrical stimulation at 15 V is more likely to activate A-δ fibers than at 5 and 10 V [31]. Therefore, electrical and mechanical stimuli may activate a number of different neurons in periphery. In the spinal cord, there are low-threshold (only responding to innocuous stimuli), high-threshold (only responding to noxious stimuli), and wide-dynamic-range neurons (responding to both), all of which receive sensory inputs from multiple primary afferents [32]. The energy consumption (e.g., oxygen and glucose) in the spinal cord should reflect a summation of all neuron types. It is thus possible that the population of neurons involved in the process of mechanical stimulation was smaller than that in the process of electrical stimulation. As a result, the energy consumption (in the spinal cord) by mechanical stimuli was smaller and less intensity-dependent than that by electrical stimuli.

### 4.2. Regional Characteristics of Hemodynamic Responses

We found clear temporal differences between the SI and spinal cord in response to mechanical or electrical stimuli (Table 2). There are two possible explanations. First, as the HbO and SO_2_ increases from the spinal cord seemed to be greater (Figure 2 and Figure 3) and longer (Table 2) than those from the SI; therefore, the spinal cord metabolism level may be greater in sensory processing. Sensory information is processed at various stages in the central nervous system, and the spinal cord is the first stage. Likely, this stage of sensory processing has less screening/filtering, and also involves ascending information to a number of supraspinal structures (e.g., the thalamus, the cerebellum, the middle brain *etc.*). As such, the sensory processing in the spinal cord may consume more energy than that in the SI—where neurons are organized with respect to specific body parts (dermatome) and only process very filtered information.

Second, there may be vasculature differences between the spinal cord and SI. Accumulating evidence indicates that during neuronal activation, a local increase in blood flow is mainly due to vasodilatation of arteries (but not veins) in the somatosensory cortex [33,34]. In our data, increases in HbO consistently occurred in both the SI and spinal cord, suggesting vasodilation of arteries during neuronal activation in sensory processing. However, changes in Hb were less consistent across regions, intensities, or modalities. It may be that the spinal cord veins act differently than the cortical veins in response to neuronal activation, and as a result, the temporal profiles of SO_2_ and HbO showed region-dependent differences.

### 4.3. Hemodynamic Signatures of Spinal Cord and SI in Response to a Long-Lasting Noxious Stimulus

The formalin test is a well-established model in study of long-lasting pain. Following a single subcutaneous injection of formalin, animals show immediate pain-behaviors (e.g., licking and elevating injected paw) for the first 5 min (Phase I), and after a 10–15 min quiescent period, they start to show pain-behaviors again for more than 1 h (Phase II) [35]. This biphasic pattern is also demonstrated by excitability of the spinal cord sensory neurons in anesthetized animals [36]. In line with these classic findings, our previous study using fNIRI in rats has found distinct hemodynamic patterns correlated with Phases I and II in a number of brain regions [37]. Similarly, in the current study we found hemodynamic responses (from the bilateral spinal cord and ipsilateral SI) during Phase I (Figure 5, Table 3), and a different response pattern (*i.e.*, sustained linear decreases in SO_2_, HbO, and HbT) during Phase II (Figure 6, Table 4). Such different response patterns between Phases I and II may underlie the well-documented biphasic physiological/behavioral pattern in the formalin test; the pattern in Phase II may indicate a hemodynamic signature of formalin-induced long-lasting pain.

It is well-accepted that an increase in blood flow to a specific brain region during functional activation is a hemodynamic signature of neuronal activation, which is referred as to hyperemia [38]. Hyperemia is primarily due to arterial dilation [33,34]. As the metabolic-rate increase is significantly less than the arterial blood influx [39], there will be a large increase in HbO and a small/no increase in Hb. Likely, regional SO_2_ (*i.e.*, HbO/(HbO+Hb)) will be elevated and approaches to arterial SO_2_ (~90%). Our observations of hemodynamics during electrical stimulation are in accordance with the canonical hyperemia notion. However, this was not the case in the formalin test (Figure 5 and Figure 6). One possible explanation for this is that the neurovascular coupling in the process of long-lasting pain may be fundamentally different from the neurovascular coupling in sensory processing at an innocuous level. When there is a much higher demand of energy consumption (e.g., in response to formalin injection), the hyperemia hypothesis (*i.e.*, arterial blood influx >> metabolic rate) may not stand. In the case of formalin injection, arteries, capillaries, and veins may all respond at a significant level due to the high metabolic-rate in the local neurovascular network. SO_2_ may no longer be merely determined by a transient boost in HbO. A small decrease in Hb (~1.5 *vs.* ~10 µM from the spinal cord; Table 3, Figure 3) can counteract an increase in SO_2_ during Phase I (non-significance; Table 3). During Phase II, all blood vessels may be fully dilated and/or reach their physiological limits for transporting blood (*i.e.*, a ceiling effect), where a change in either HbO or Hb cannot meet the demand of heavy energy consumption in the process of the long-lasting pain (*i.e.*, arterial blood influx << metabolic rate); only a decrease in SO_2_ can reflect the ongoing energy consumption.

Hindlimb injection of formalin is only an animal model for long-lasting pain. To the best of our knowledge, there is neither any human data characterizing formalin-induced pain, nor an equivalent type of persistent pain in humans that lasts for one hour or so. Thus, there is a scarcity of studies that link between the common SO_2_ assessment in human clinic [40,41] and the physiology of long-lasting pain. Nevertheless, both human and animal data using hyperbaric oxygen therapy suggests that a systemic increase in SO_2_ can alleviate chronic pain (note that chronic pain is associated with a neuropathological condition that often lasts for days and beyond) [42,43]. Together with our formalin results, the hyperbaric-oxygen-therapy-induced analgesic effect may indicate a causal relationship between persistent pain and a decreasing SO_2_ in associated regions in the central nervous system. That is, a decreasing SO_2_ may underlie an aberrant interaction between neurons and vascular activity; this aberrant interaction may play a role in pain processing.

### 4.4. Regional Characteristics of Light Scattering

Our measurements in the baseline period as well as in response to stimuli are in line with standard practices in the field—the white matter has a greater light-scattering effect than grey matter [44]. Because our probes were placed above the dorsal surface of rat SI and above the dorsal column of rat spinal cord, our baseline measurements (μ_s_′: SI < spinal cord; Table 1) should mainly characterize the light-scattering difference between the white matter and grey matter. In response to stimuli, the changes in light scattering appeared to be complex; both positive and negative light-scattering changes have been found in rat somatosensory cortex during forepaw stimulation [17]. Our results showed a decrease in light scattering only in a limited number of stimulation and measurement conditions (Figure 3, Figure 5 and Figure 6). Stimulation-induced light-scattering change appeared to be dependent on time, region, intensity, and modality.

### 4.5. Anesthesia

Pentobarbital anesthesia—known to depress cardiovascular activity and respiration [45]—was used, because an *in vivo* assessment in awake rats using our customized device is extremely challenging. The use of anesthesia completely ruled out such confounds as motion artifacts and behavioral states, and ensured precise and consistent locations of scanned areas of interest over a long period of time. In short, the use of anesthesia allowed us to inspect unique patterns of response to nociceptive/noxious stimuli. In addition, as general anesthesia appears not to affect the early onset of evoked responses of the central nervous system [46,47], our results under pentobarbital anesthesia in rats may to some extent parallel some genuine characteristics of neurovascular response to somatosensory/nociceptive stimulation in the awake state. However, because general anesthesia alters arousal states [48], and pain perception consists of sensory, affective, and cognitive aspects, an interpretation of our results should be cautious in comparison to human data derived from neuroimaging techniques.

## 5. Conclusions

A gradual decrease in SO_2_ appeared to be a unique pattern for formalin-induced sustained pain, which may be a biomarker candidate for long-lasting pain. We also found changes in hemodynamic parameters and light scattering in rat spinal cord and SI during brief peripheral stimulation. Patterns of these changes (e.g., amplitude, duration, lateralization, increase or decrease) in a single parameter did not show a distinction between innocuous and noxious stimulation. Instead, patterns of these changes depended on time, region, stimulus intensity, and modality. We expect that a multiple-parameter strategy with careful consideration of combining factors such as regions and stimulus modality may be more effective in detecting pain using neuroimaging technologies.
